# Relationship between Emotional Intelligence, Educational Achievement and Academic Stress of Pre-Service Teachers

**DOI:** 10.3390/bs11070095

**Published:** 2021-06-23

**Authors:** Inmaculada García-Martínez, Eufrasio Pérez-Navío, Miguel Pérez-Ferra, Rocío Quijano-López

**Affiliations:** 1 Department of Didactics and School Organization, University of Granada, 18071 Granada, Spain; igmartinez@ugr.es; 2 Department of Pedagogy, University of Jaén, 23071 Jaén, Spain; epnavio@ujaen.es; 3 Department of Science Education, University of Jaén, 23071 Jaén, Spain

**Keywords:** emotional intelligence, academic stress, pre-service teachers, achievement rating

## Abstract

Emotional intelligence (EI) and stress are constructs that often characterize the teaching profession and are inversely related. There is evidence in the literature that suggests the importance of teachers working on EI in order to learn coping strategies and improve their teaching practices. This descriptive and correlational study had the purpose of examining the social–emotional profile of future teachers based on their EI and academic stress levels in order to provide guidance for future stressful situations that will affect their future professional development. For this purpose, we used a random sampling for convenience in a university population enrolled in degrees of education at Andalusian universities (Spain), getting a sample of 1020 pre-service teachers. The results pointed to a superiority in EI, academic stress, and academic achievement in favor of females compared to males. The relationship among EI, academic stress, and student teachers’ achievement was demonstrated. Furthermore, some components of EI were positioned as important factors to improve student achievement and reduce academic stress. Once the high incidence of these constructs on academic achievement was confirmed, the importance of developing EI and coping and stress skills training programs, aimed at improving academic success and their subsequent professional development, was demonstrated.

## 1. Introduction

### 1.1. University Training

The university stage is characterized by constant changes that impact on the physical and psychological well-being of university students [1]. Access to university depends on achieving the required “cutoff” score for the university degree they want to study. As a result, students tend to acquire study habits and “measure” their efforts according to the degree for which they wish to enroll in at university. In the case of education degrees in Spain, the access mark is usually low compared to other degrees, such as health sciences [2]. This means that many candidates for education have not learned effective study habits, nor have they developed enough planning and time management strategies to cope with the subsequent studies [3]. In addition, unlike previous educational stages, students see how their expected academic demands multiply exponentially. Likewise, the teaching and learning processes are changed with respect to earlier stages, and they tend to fit into academic courses rather than outgrow them. [4]. While in secondary education, the teacher plays a key role in guidance and the development of more direct instruction, self-learning and students’ ability to build their own learning autonomously are given priority at university [5]. Added to this situation is that universities place emphasis on academic excellence. The world of work is increasingly demanding, and it requires highly qualified, multifaceted, and competent professionals capable of coping with an ever-changing society. It is not enough just to have an academic diploma; competitiveness is encouraged to achieve excellence [6]. In this regard, universities have taken up these demands and included these parameters in their curriculum. Also, governments have revised their educational grant system to seek out the best recipients.

### 1.2. Academic Stress in the University Context

Academic achievement and excellence are becoming increasingly important for students in general, and students of education degrees in particular, since their permanence in the university depends, in many cases, on student grants linked to their academic achievement. This fact produces significant stress for university students because they need to obtain high scores in order to finish their studies and seek better employment in their future [7].

In this period, which coincides with the transition from adolescence to adulthood, there are other personal, economic, and social factors that appear and affect personality, understanding of life, and socioemotional balance [8,9]. When students become adults and move to the university, they may become geographically independent because they leave their families to move to the city, which is another great challenge that many students have to face [10]. Thus, the transition to the university stage includes changes in the social, academic, and personal spheres of the individual, which tends to generate stress and anxiety in university students [11,12]. A study developed by Meehan and Howells [13] with university students found that the perceived sense of belonging was one of the most important factors when it came to evaluating their time at a university. The academic staff they were with and the type of studies they were involved in were further issues to be taken into account. These findings confirm the importance of the social and personal dimension in achieving emotional and psychological balance [14]. This stress tends to be reduced over the course of university. Although the personal and social dimensions become more established as a result of students’ adaptation in their new residences and the creation of emotional relationships with peers [15], new challenges appear related to the academic environment and their imminent entry into the world of work, generating high levels of stress in students [16].

It is precisely at the university stage where all these academic–professional changes happen and, combined with growing social demands, this is what has raised interest in analyzing the stress that these situations cause in students [17]. Academic stress includes the traditional dichotomy that goes along with the definition of stress. Firstly, it includes the stressors that produce that condition, and secondly, it focuses on the behavioral and physiological reactions that these stressors trigger individually [18]. Thus, stress arises when, under academic stressors (performance and academic excellence), students’ subjective experiences of stress and the feeling of loss of control when faced with new challenges to overcome come into play [19]. Some studies have pointed out three major groups of academic stressors: (a) aspects related to assessment, (b) increased “workload”, and (c) aspects linked to the instructional processes, such as methodology or the teacher–student relationship [20]. These issues produce a physiological and psychological response as stressful situations or stressors. Consequently, the physical and psychological well-being of the individual may be impaired. As a result, this may affect their self-concept, psychological balance, or food intake, or cause the appearance of addictions [21,22]. Stress is defined by Lazarus and Folkman [23] as a particular relationship between individuals and their environment, which is regarded by said individuals as imposing or beyond their resources. Academic stress experienced by university students refers to factors related to the academic environment such as coursework, group projects, and organizational involvement, as well as perceptions, attitudes, and behaviors towards academic demands [24]. Academic stress appears when students perceive that the resources they have are not enough to cope with situations that are challenging for them. This situation of distress causes anxiety responses that affect their successful development. Academic stress negatively affects students’ personal, emotional, and physical well-being [25], as well as affecting their learning and achievement standards [26]. In addition, Watson and Watson [27] examined how much EI and coping skills predicted academic stress in first-year university students, and they found that the absence of emotional intelligence was a good predictor of stress. Similarly, recent studies show a negative relationship between EI and academic stress [1,28,29].

### 1.3. Emotional Intelligence

A variety of stress-modulating variables have been identified as “buffers” to stop the adverse effects of stress on students’ health. One of these buffers could be the existence of academic tutoring, which, together with the presence of high EI levels, plays a critical role in personal self-regulation and effective stress management [30]. Regarding this first possibility, several investigations have pointed out the importance of academic tutoring to support and promote personal and professional growth oriented to success [31,32]. On the other hand, EI as the ability of individuals to know, explain, understand, and manage their own and other people’s emotions has become a desirable skill to achieve as well as an effective measure of physical and psychosocial health [33,34]. However, the analysis of emotional intelligence is rather challenging. In the literature, two different conceptions are identified: emotional intelligence as an ability and emotional intelligence as a trait. Furthermore, the conceptualization of the EI framework has implications for measurement. Ability theorists tend to suggest that EI is best measured by performance tasks, whereas trait theorists believe that EI can be assessed by self-report tools that assess the perceived efficacy of emotional processing. Moreover, the relationship between trait and ability assessments of EI and academic outcomes is not uniform.

In this study, we assume the most accepted conceptualization of EI, which describes it as the ability of individuals to perceive, assimilate, understand, and regulate their own and other people’s emotions [35]. In this manner, emotional intelligence may be viewed as the constellation of personality dispositions that affect how people process emotional information. Understanding the EI construct requires the recognition of five areas divided into social and personal competences: self-awareness, self-regulation, self-motivation, social awareness, and social skills [36]. Taking this into account, a person is considered emotionally intelligent when they have internalized and dominated these skills. Unlike other types of intelligence, such as intelligence dependent on IQ, EI can be developed with suitable training [37]. This fact has motivated an important deployment of research oriented to learning and training based on EI and social skills that lead to professional and academic success [38]. In fact, in the literature, there are many studies on EI’s modulating role to reduce the effects of stress [39,40]. The growing interest of the world of work and society in obtaining emotionally competent professionals has encouraged universities to carry out studies aimed at analyzing and promoting EI among students [41,42]. This ability is especially important for those whose future professional practice will be in social contexts. Specifically, the teaching profession is one of the professions with the greatest emotional burnout, since members are exposed to high levels of stress as a result of trying to cope with the growing demands they have to face in their professional lives [43,44,45]. A good way to prevent these situations is to find out the emotional profile of aspiring teachers in order to make an assessment that can act as the basis for the development of actions designed to improve certain social and emotional skills and achieve greater employability in the world of work [46].

### 1.4. Our Study

Given the lack of studies on EI, academic stress, and achievement for the teacher-in-training population, we sought to analyze these variables with a large sample in order to understand these relationships. In this regard, this study assumed the importance of having high levels of emotional intelligence in order to cope successfully with academic stress. In addition, the student’s average mark to date was included as one factor that affects these variables. Although the focus is on teacher training, this study will provide the basis for future research with in-service teachers.

Therefore, this research had the purpose of examining the emotional abilities of future teachers based on their EI and academic stress levels in order to provide guidance for future stressful situations that will affect their future professional development. For this purpose, attention was paid to the variable of gender in order to know if there are differences in emotional intelligence based on gender. Likewise, we intended to analyze the relationship among EI levels, academic stress, and academic achievement. Taking the above into account, this study addressed the following research questions:

- What are the levels of emotional intelligence and academic stress of pre-service teachers?
- Are there differences in the measured constructs according to gender?
- What is the relationship among emotional intelligence, academic stress, and academic performance of pre-service teachers?

## 2. Materials and Methods

### 2.1. Subjects and Design

A descriptive, cross-sectional and non-experimental research project was carried out with 1020 university students studying education degrees. In relation to gender, it was found that 75.78% were women and 24.21% were men. Ages ranged from 17 to 50 years, with an average age of 21.52 and SD = 4.44. In relation to the degrees subjects studied, 42.8% were enrolled in Primary Education, 30.7% in Early Childhood Education, 14.4% in Social Education, 10.4% in a Master’s degree in Teaching, and 1.7% in Pedagogy. In relation to course level, it was found that 57.2% were enrolled in the first year, 9.9% were in the second, 18. 7% in the third and 14.2% in the fourth. Finally, with regard to the region, it was found that 56.5% studied in Jaén, followed by Granada (13.1%), Córdoba (10.4%), Sevilla (5.5%), Cádiz (5.4%), Málaga (4.2%), Almería (2.7%), and Huelva (2.2%).

### 2.2. Instruments

Three different types of instruments were used in this research project (they are available in the Appendix A). The first was an ad hoc questionnaire where undergraduates indicated their sex, age, city, studies, and academic year they were in. Finally, they were asked for their average mark to date of their degree (the overall average mark obtained in courses by the student).

As the second instrument, we used Wong and Law’s Emotional Intelligence Scale [47]. This scale is composed of 16 short sentences used to evaluate four dimensions: Self-Emotion Appraisal (SEA), Others’ Emotion Appraisal (OEA), Use of Emotion (UOE), and Regulation of Emotion (ROE). SEA refers to the perceived ability to appraise emotions; OEA addresses people’s ability to appreciate the emotions of others; UOE concerns people’s ability to use their emotions effectively; and ROE is the ability of people to manage their emotions and adjust them to the situation they were experiencing. Respondents were asked to rate their agreement with the sentences on a five-point Likert scale ranging from 1 (strongly disagree) to 5 (strongly agree). We used the Spanish version of Extremera et al. [48], which has shown adequate validity and reliability in Spanish contexts (α = 0.91). The internal reliability of this scale in the present study for all items was α = 0.918.

Third, we used the SISCO Inventory of academic stress [49]. This instrument includes 37 items with five answer options, ranging from 1 (never) to 5 (always). It identifies three categories, symptoms, stressors, and coping strategies, with a reliability of 0.9. Symptoms refer to the set of physical, psychological, and behavioral reactions that a person presents in a stressful situation. Stressors, on the other hand, are those circumstances, conditions. or situations that generate stress in people. A range of academic situations involving stress is presented with the aim of analyzing how stressful they are for students. It also measures the frequency at which people experience a set of psychological, physiological, and behavioral reactions when faced with these situations. Finally, the instrument evaluates the use of a variety of coping strategies used to address stress. In the present study, the overall internal reliability consistency for this questionnaire was α = 0.916.

### 2.3. Procedure

The instruments were administered using a Google Form tool. The researchers attended the classes of the potential participants to explain the purpose of the research. In those cases where this was not possible, the teaching staff was informed to transfer the information to their students and provide them the link to complete the questionnaire. In all cases, the emails of the researchers were provided for contact in case of doubts or need for further information. Also, all participants had to be student teachers as an inclusion criterion. 1150 participants were invited to participate, of whom 119 declined to participate or did not answer, and 16 questionnaires had to be removed for not being properly completed. Participation in the study was completely voluntary, in accordance with the Declaration of Helsinki in 1975 and its next adjustment from Brazil in 2013 [50], respecting the national legislation for clinical trials (223/2004 Law from 6 February), biomedical research (14/2007 Law from 3 July), and participants’ confidentiality (15/1999 from 13 December) and the Human Research Ethics Committee of the University of Jaén (code OCT.20/1.TES), regulated by Andalusian Decree 439/2010 of 14 December.

### 2.4. Data Analysis

For data analysis, the statistical software IBM SPSS 25.0. (International Business Machines Corporation, Armonk, NY, USA) was used in order to establish the values of the basic descriptors (means and frequencies). The magnitude of the differences of effect size was determined using Cohen’s standardized measure d and was interpreted as zero (0–0.19), low (0.20–0.49), moderate (0.50–0.79), or high (≥0.80). Therefore, for each effect size, the 95% confidence interval (95% CI) was calculated. In order to understand differences between two correlations, the effect size was calculated with Cohen’s q. In addition, regression was used to establish the relationships among the variables that made up the correlational model for both groups (males and females). In this case, the causal explanations of the endogenous variables were made considering the observed associations between the indicators and the reliability of the measurements. Thus, the measurement error of the observed variables was included and could be directly controlled and interpreted as multivariate regression coefficients.

The differences between variables of categorical and interval type were analyzed using Student’s t-test. Similarly, the Bonferroni test was applied to verify intergroup differences in all the variables. We also performed linear regression analysis to study the association between EI, AS (independent variables), and student achievement (dependent variable), adjusted for gender.

## 3. Results

### 3.1. Descriptives

The descriptive results (Table 1) show how there were statistically significant differences in relation to average mark to date of their degree studies between female (8.87 ± 1.68) and male (8.25 ± 1.17) (*p* = 0.021) subjects through the t-test. Likewise, for EI, women scored higher than men, with statistically significant differences in all dimensions (*p* ≤ 0.000). Regarding academic stress, statistically significant differences were found in favor of women in the symptoms dimension (2.82 ± 0.73 vs. 2.51 ± 0.74) (*p* ≤ 0.000) and in the stressors dimension (3.59 ± 0.60 vs. 3.32 ± 0.66) (*p* ≤ 0.000). No differences by gender were found for the coping strategies dimension.

### 3.2. Correlations

Table 2 shows the correlations between EI and academic stress in relation to females. The strong positive correlations between OEA and stressors (r = 0.900), between ROE and symptoms (r = 0.818), and between UOE and symptoms (r = 0.753), in the case of females, stand out. Likewise, there are also correlations between UOE and ROE (r = 0.581) and between UOE and stressors (r = 0.477). It also stands out how there is a negative correlation between SEA and symptoms (r = −0.240). Positive relationships were also found between stressors and symptoms (r = 0.356) and between stressors and ROE (r = 0.266). Relationships were also found between symptoms and OEA (r = 0.289) and between OEA and UOE (r = 0.381). The rest of the correlations found are weak.

The correlations between EI and academic stress in the case of males (Table 3) are slightly higher, highlighting the strong positive correlations between OEA and stressors (r = 0.915), between ROE and symptoms (r = 0.830), and between UOE and symptoms (r = 0.762). Likewise, there are medium correlations between UOE and ROE (r = 0.595) and UOE and stressors (r = 0.479). In addition, positive relationships were found for the stressors dimension with symptoms and ROE (r = 0.350; r = 0.273). The negative correlation between SEA and symptoms (r = −0.215) and ROE (r = −0.286) stands out. The remaining correlations found are weak.

### 3.3. Regression Analysis

Linear regression analyses were performed to check the association between the gender of participants with their average mark to date in their degree, WLEIS, and SISCO categories. The regression was also carried out differentiating between female and male (Table 4). For the predictive models in relation to female sex, average mark to date in degree was a predictive variable (β = 0.005; *p* ≤ 0.000), which explains 39% of the variance of the response variable. In relation to the EI, the dimension SEA (β = 0.010; *p* = 0.021) explains 32% of the variance; UOE (β = 0.007; *p* = 0.038) explains 48% of the variance; and ROE (β = 0.039; *p* = 0.021) explains 64% of the variance. Likewise, for the dimensions of academic stress, it was found that symptoms (β = 0.027; *p* ≤ 0.000) and stressors (β = 0.022; *p* = 0.028) explain 10% and 33% of the variance of female responses, respectively.

On the other hand, in the case of males, average mark to date in degree was a predictive variable (β = −0.014; *p* = 0.018) that explains 30% of the variance of the response variable. Regarding EI, the dimension SEA (β = −0.016; *p* = 0.021) explains 57% of the variance; OEA (β = 0.150; *p* = 0.016) explains 57%; UOE (β = 0.036; *p* = 0.024) explains 21%; and ROE (β = 0.009; *p* = 0.014) explains 51%. With respect to the dimensions of academic stress, it was found that symptoms (β = −0.085; *p* = 0.035) and stressors (β = 0.001; *p* = 0.041) explain 15% and 36% of the variance of male responses, respectively.

## 4. Discussion

Our findings provide an analysis of the psychosocial profile of future teachers and their relationship to academic performance based on EI, AS, and student teachers’ achievements to date. More specifically, this study sought to answer three research questions. In this section, each of these questions is addressed:

- What are the levels of emotional intelligence and academic stress of pre-service teachers?

Based on the results obtained, it was determined that future teachers tend to perceive the emotions of others better than their own emotions, although these two dimensions of emotional intelligence are the highest. In relation to the use of emotions and the regulation of emotions, medium values were detected, although lower than those for the recognition of emotions. Other studies have also found that emotional regulation is the lowest dimension [51].

In relation to academic stress, it was found that future teachers perceive different situations as stressful, although they do not have a wide variety of symptoms. In terms of coping strategies, the values found were moderate, a finding that can be understood by the emotional intelligence scores. In this line, a study by Alva et al. [52] found that half of the students studied presented low levels of academic stress, 8.2% had medium levels, and 39% showed high levels, and that the most used coping strategy was “assertive ability”.

- Are there differences in the measured constructs according to gender?

In relation to the average mark to date of their degree, significant differences were found in favor of women, consistently with other studies [48,53,54]. In the literature, there is evidence of a higher order of women in academic achievement in this area, and this variable is related to greater involvement, critical thinking, and vocation, especially in educational degrees [55].

Examining the psychosocial profile by gender, it was found that females tend to be more emotionally intelligent than males. This was revealed by the results obtained for the four dimensions that constitute the EI construct, where statistically significant differences were found. These findings are consistent with research on EI in the university population [56,57], and even in the adult population [48].

In terms of academic stress, we found higher scores for females over males, consistently with other studies [58,59]. In our case, statistically significant differences were found in favor of females in the symptoms and stressors dimensions and slightly in favor of males in the coping strategies. These results are similar to those obtained in a study by Manrique-Millones et al. [60], where females scored higher than males in the stressors and symptoms subscale and no gender differences were found regarding coping subscale.

- What is the relationship among emotional intelligence, academic stress, and academic performance of pre-service teachers?

In the correlational analysis, strong relationships were found between OEA and stressors, between ROE and symptoms, and between ROE and symptoms for both genders [61].

Our findings are explained by the fact that people who have higher levels of understanding of others’ emotions tend to have a greater predisposition to suffer stress [62], as they may appropriate what others feel. These cases can lead to lower academic achievement [63]. In contrast to the present study, previous research on EI and stress has found a strong inverse relationship between these constructs [1] and a direct relationship between EI and mental health [64]. Thus, studies such as Kassim et al. [65] have maintained that students who are emotionally intelligent are more capable to manage stress and that this is demonstrated in their academic achievement. Other studies confirm this trend, considering EI a “buffer” for stress and its adverse health effects [66]. Similarly, a study by Enns et al. [12] demonstrated the mediating effect of coping on the relationship between EI and stress in the university population. Other studies have also pointed out the relationship between EI and coping, claiming that more emotionally intelligent people tend to have a greater variety of coping resources and adaptive behaviors, positively affecting their psychosocial balance and health.

Another aspect to be evaluated was the relationship among EI, academic stress, and relation to academic achievement [60]. This enquiry is aligned with research that places emotional intelligence as a predictor of academic achievement and consequent success in the world of work. For example, a study by Kuk et al. [67] with university students argues for the importance of working on EI in order to reduce the intensity of stress symptoms and to better manage students’ professional future in social services. From a quasiexperimental study, they demonstrated the effectiveness of psychological workshops in this work, finding that the greatest predictor of emotional intelligence was the ability of students to control depression and that acceptance of emotion was particularly important in problem solving, coping in difficult situations, and forgiveness with respect to internal experiences. Along this line, but with practicing teachers, Mérida-López et al. [43] argued for the importance of developing emotional intelligence to reduce the stress that usually follows the teaching profession, finding that EI and engagement are related to the level of teachers’ commitment, which is necessary to become an effective teacher.

Similarly, other studies have pointed out the importance of working on emotions for pre-service teachers, as novice teachers tend to show more negative attitudes towards challenging situations, especially when they do not have the emotional resources to manage these emotions. In turn, EI is related to occupational engagement and academic motivation [17], so it is a priority to include emotional skill training programs to reduce pre-service teachers’ stress and empower them to cope with the challenges expected to be faced.

## 5. Conclusions

This research had the purpose of examining the social–emotional profile of future teachers based on their EI and academic stress levels and the relationship of these factors with academic achievement. The analysis of this educational group by gender confirmed what has already been found in the literature about the higher scores of females in all dimensions of EI over males. This superiority was also replicated when academic stress was analyzed by gender. These aspects can be decisive in the analysis of students’ academic achievement; in fact, the results obtained determined that females had higher average grades than males. Once the high incidence of these constructs on academic achievement is confirmed, the importance of developing EI and coping and stress skills training programs, aimed at improving academic success and their subsequent professional development, was demonstrated.

### 5.1. Limitations

This study had certain limitations that must be considered, prior to reading and stating the findings found.

Firstly, the research design itself may have been a bias to interpreting the findings. Longitudinal designs will allow a better understanding of the behavior of the variables analyzed. Likewise, the implementation of training programs in emotional skills will enable analysis of the impact of instruction in emotional intelligence on the socioemotional profile of future teachers, as a preliminary step to improve their professionalization. The second limitation was related to the sample. Despite the fact that the proportion of men and women in the sample is consistent with the predominant trend for the university population assigned to educational degrees in Spain, the superiority of females over males may be a bias. Furthermore, self-assessment questionnaires were the instruments used. Further studies could complement the instruments used with others, which would provide a more comprehensive diagnosis of the variables examined. Similarly, the use of the average mark to date for these student teachers as the only indicator of academic achievement may be insufficient. This limitation could be corrected in the future by including items related to study habits, engagement, and time spent preparing academic assignments and studying.

### 5.2. Further Research and Practical Implications

Analysis of the psychosocial tendencies of pre-service teachers is an essential element. If we know what teachers are like, we may develop interventions and programs aimed at improving EI and coping strategies that can reduce the high levels of stress and burnout associated with the teaching profession. In this regard, further research will include other factors identified in the literature as important to teacher professional development, such as resilience or personality. In addition, longitudinal research, with instructional programs based on emotional skills, coping strategies, and resilience, should be conducted to assess the potential impact of these factors on future teachers.

Preventing high levels of anxiety and depression in pre-service teachers requires training that is tailored to the demands they will experience in their future professional development. The design of this type of program requires a diagnosis of what future teachers are like. To this end, studies of this type contribute positively to the achievement of this objective, as they provide a general overview of the situation and of which aspects need to be examined in order to continue with the improvement of these professionals.

## Figures and Tables

**Table 1:** Average mark to date in degree, emotional intelligence, and academic stress according to gender.

Variable	Male		Female		Levene Test		Sig. (Bilateral)	ES (d)	95% CI
	M	S.D.	M	S.D.	F	Sig.			
Average mark to date in degree	8.25	1.17	8.87	1.68	0.27	0.24	0.021 *	0.103	(7.61; 9.52)
SEA	11.23	2.75	12.03	2.62	17.09	0.70	0.000 *	0.161	(11.68; 12.37)
OEA	13.51	3.08	14.68	2.84	30.30	0.45	0.000 *	0.114	(13.13; 14.89)
UOE	10.51	3.18	12.59	3.29	75.22	0.11	0.000 *	0.119	(10.12; 12.82)
ROE	10.85	3.45	12.17	3.54	26.11	0.17	0.000 *	0.281	(10.42; 12.42)
Symptoms	2.51	0.74	2.82	0.73	33.66	0.54	0.000 *	0.328	(2.41; 2.87)
Stressors	3.32	0.66	3.59	0.60	33.80	0.38	0.000 *	0.157	(3.24; 3.63)
Coping strategies	3.00	0.61	3.01	0.60	0.10	0.37	0.742	0.076	(2.92; 3.06)

Note: * *p* < 0.05; SEA: Self-Emotion Appraisal; OEA: Others’ Emotion Appraisal; UOE: Use of Emotion; ROE: Regulation of Emotion.

**Table 2:** Bivariate correlations between EI and academic stress categories for females.

	SEA	OEA	UOE	ROE	Symptoms	Stressors	Coping Strategies
**SEA**	1	−0.057	−0.158 *	−0.150 *	−0.240 *	−0.067	0.197 *
**OEA**		1	0.381 *	0.227 *	0.289 *	0.900 *	0.113 *
**UOE**			1	0.581 *	0.753 *	0.477 *	0.069
**ROE**				1	0.818 *	0.266 *	0.128 *
**Symptoms**					1	0.356 *	0.061
**Stressors**						1	0.143 *
**Coping strategies**							1

Note: * *p* < 0.05; SEA: Self-Emotion Appraisal; OEA: Others’ Emotion Appraisal; UOE: Use of Emotion; ROE: Regulation of Emotion.

**Table 3:** Bivariate correlations among EI and academic stress categories for males.

	SEA	OEA	UOE	ROE	Symptoms	Stressors	Coping Strategies
**SEA**	1	−0.019	−0.064	−0.186 **	−0.215 **	−0.059	0.128 *
**OEA**		1	0.299 **	0.181 **	0.204 **	0.915 **	0.146 *
**UOE**			1	0.595 **	0.762 **	0.479 **	0.064 *
**ROE**				1	0.830 **	0.273 **	0.097 **
**Symptoms**					1	0.350 **	0.049
**Stressors**						1	0.142 **
**Coping strategies**							1

Note: * Significant correlation at level 0.05; ** Significant correlation at level 0.01; SEA: Self-Emotion Appraisal; OEA: Others’ Emotion Appraisal; UOE: Use of Emotion; ROE: Regulation of Emotion.

**Table 4:** Bivariate correlations among EI and academic stress categories for males.

Variables	Standardized *β*	*t*	*p*	R^2^	Adjusted R^2^
**Average mark to date in degree**					
Female	0.005	−3.214	0.000	0.421	0.392
Male	−0.014	−0.343	0.018	0.352	0.304
**SEA**					
Female	0.010	−1.571	0.021	0.327	0.320
Male	−0.016	−0.343	0.001	0.582	0.576
**OEA**					
Female	0.006	−1.772	0.147	0.211	0.205
Male	0.150	0.594	0.016	0.154	0.149
**UOE**					
Female	0.007	0.038	0.030	0.515	0.487
Male	0.036	0.044	0.024	0.223	0.219
**ROE**					
Female	0.039	−0.789	0.021	0.651	0.644
Male	0.009	−0.188	0.014	0.524	0.519
**Symptoms**					
Female	0.027	0.945	0.000	0.110	0.106
Male	−0.085	0.454	0.035	0.164	0.158
**Stressors**					
Female	0.022	0.595	0.028	0.345	0.338
Male	0.001	0.922	0.041	0.267	0.260
**Coping strategies**					
Female	0.026	0.186	0.853	0.113	0.097
Male	−0.014	1.263	0.590	0.287	0.281

Note: *p* < 0.05; SEA: Self-Emotion Appraisal; OEA: Others’ Emotion Appraisal; UOE: Use of Emotion; ROE: Regulation of Emotion.

## Data Availability

Data are available on justified request to the corresponding author.

## Appendix A. Instruments Used in This Research Translated to English

**Wong Law Emotional Intelligence Scale (WLEIS-S)**
**Self-Emotion Appraisal, SEA**
I have a good sense of why I have certain feelings most of the time
I have good understanding of my own emotions
I really understand what I feel
I always know whether or not I am happy
**Others’ Emotion Appraisal, OEA**
I always know my friends’ emotions from their behavior
I am a good observer of others’ emotions
I am sensitive to the feelings and emotions of others
I have good understanding of the emotions of people around me
**Use of Emotion, UOE**
I always set goals for myself and then try my best to achieve them
I always tell myself I am a competent person
I am a self-motivating person
I would always encourage myself to try my best
**Regulation of Emotion, ROE**
I am able to control my temper so that I can handle difficulties rationally
I am quite capable of controlling my own emotions
I can always calm down quickly when I am very angry
I have good control of my own emotions

**SISCO Inventory Items**
Competition with peers
Overload of homework and schoolwork
Teacher’s personality and character
Teacher’s assessments (exams, essays, research papers, etc.)
The type of work teachers ask you to do (topic consultation, worksheets, essays, concept maps, etc.).
Failure to understand the topics covered in class Participation in class (answering questions, presentations, etc.)
Limited time to do the work
**Physical reactions**
Sleep disturbances (insomnia or nightmares)
Chronic fatigue (permanent tiredness)
Headaches or migraines
Digestion problems, abdominal pain or diarrhea
Scratching, nail biting, rubbing, etc.
Drowsiness or increased need for sleep
**Psychological reactions**
Restlessness (inability to relax and be calm)
Feelings of depression and sadness (feeling down).
Anxiety, anguish or desperation.
Problems with concentration
Feelings of aggression or increased irritability
**Behavioral reactions**
Conflict or tendency to argue or dispute
Isolation from others
Unwillingness to do schoolwork
Increased or decreased food consumption
**Coping strategies**
Assertive skills (defending our preferences, ideas or feelings without harming others)
Making a plan and executing your tasks
Self-praise
Religiousness (prayers or attending mass)
Seeking information about the situation
Ventilation and confidences (verbalization of the situation)
